# Intense up-conversion luminescence from Dy^3+^-doped multi-component telluroborate glass matrix: Role of CuO nanoparticles embedment

**DOI:** 10.1016/j.heliyon.2023.e15906

**Published:** 2023-05-04

**Authors:** I. Abdullahi, S. Hashim, M.I. Sayyed, S.K. Ghoshal

**Affiliations:** a Department of Physics, Universiti Teknologi Malaysia, 81310, Skudai, Johor, Malaysia; b Department of Physics Federal University Gusau, Zamfara State, Nigeria; c Department of Physics, Isra University, Amman, Jordan

**Keywords:** Up-conversion, Dysprosium, Copper oxide, Judd-Ofelt, Energy transfer, Radiative properties

## Abstract

This paper reports an intense up-conversion luminescence from Dy^3+^-doped strontium-telluro-alumino-magnesium-borate glasses for the first time. The samples were made via the melt-quenching method and characterized to determine the influence of various CuO nanoparticles contents change on their up-conversion emission traits. Absorption spectral data were used to calculate the Judd-Ofelt intensity parameters. The sample without CuO nanoparticles revealed two intense photoluminescence up-conversion emission peaks at 478 and 570 nm. In addition, CuO nanoparticles-activated sample displayed about 1.4-fold up-conversion emission intensity improvement due to strong light absorption in the visible to the infrared region at 799 nm excitation. The stimulated emission cross-section of the CuO nanoparticles-activated glasses was increased from 102.4 × 10^−23^ to 1301.1 × 10^−23^ cm^2^ (nearly 10-fold amplification) while the branching ratio was reduced to 66.9%. Thus, CuO nanoparticles as an additive in the current glass matrix enhanced the up-conversion emission and strengthened the associated nonlinear optical properties. CIE 1931 color matching revealed the influence of CuO in modifying the up-conversion color coordinates, thereby improving the white color purity. The achieved up-conversion emission coupled with the color tunability of the proposed glasses may be advantageous for the up-conversion UV tunable laser making.

## 1 Introduction

Up-conversion emission is a nonlinear optical phenomenon based on the anti-Stokes shift, wherein photons with lower energy produce the luminescence emission at higher energy [[1], [2], [3]]. Due to their distinct and substantial nonlinear optical properties, rare earth ions (REIs) doped systems are the dominant up-conversion materials applied in solid-state lasers, digital telecommunications, fingerprint technology, advanced optical sensing, and digital display systems [[4], [5], [6]]. Photon up-conversion process can be mediated by either the excited state absorption (ESA), energy transfer up-conversion (ETU), or photon avalanche (PA) [7,8] mechanisms. While ESA entails purely REIs of the same element, ETU and PA may also involve REIs of different elements. Recent studies have demonstrated the ESA [9], ETU [10], and PA [11] processes. These previous works asserted the suitability of the obtained up-conversion emissions for application in broadband amplification, fiber lasing, and fingerprinting technology.

Various studies hinted the success of an up-conversion system design depends on the REIs-host combination. In this context, an efficient and reliable ion-host combination is needed [12]. The REIs-glass combination proved to be efficient for up-conversion luminescence. The infrared to visible or ultraviolet strong up-conversion emission of REIs is due to their long-lived metastable electronic energy levels, a consequence of the spin-forbidden nature of the intra-4f transitions. Among the REIs, Dy^3+^ is known to increase the compactness of glass host by filling the voids within the structural network, act as an absorption center for many transitions, exhibit blue solid and yellow emissions in the visible region, and act as an efficient sensitizer in REIs codoped systems [[13], [14], [15]]. However, probably due to the scarcity of highly intense excitation sources matching the specific excitation wavelength essential for Dy^3+^, very few studies explored the up-conversion mechanism of dysprosium-doped systems [16]. The few available mostly relate to crystal codoped systems with Dy^3+^ as a sensitizer. For example, Yang et al. [17] reported the up-conversion luminescence of Er^3+^-Dy^3+^ co-doped CaF_2_ phosphor under 900 nm excitation. They found that Dy^3+^ acts as a sensitizer and can make the orange-yellow-green up-conversion color tunable by modifying the Er^3+^/Dy^3+^ ratio. Moreover, the up-conversion emission of Dy^3+^/Yb^3+^ co-activation in glass ceramic hosts incorporating strontium fluoride nanocrystals upon 980 nm excitation was reported [18]. Details of the up-conversion mechanisms and mediation of the perceived luminescence were proposed, and the results show that the glass system can be used for white-light imaging technology. Again, it was reported that Dy^3+^/Yb^3+^ glass systems revealed feeble up-conversion blue, yellow and red emissions upon 976 nm excitation [19]. Nonetheless, none of these reports contains the up-conversion mechanism details of Dy^3+^ singly doped glass systems.

Due to their ability to optimize the nonlinear optical properties of rare-earth-doped systems for diverse applications, the embedment of optically active nanoparticles is being adopted for up-conversion emission enhancement [20]. For example, Dousti et al. reported a significant enhancement of Er^3+^ up-conversion emission stimulated by Ag Nps [21]. The presence of Au Nps with an average diameter of 20 nm caused a 30-fold luminescence enhancement. Similarly, Rajaramakrishna et al. revealed in their work the luminescence enhancement mediated by Ag Nps in REIs doped silicate glass system [22]. The researchers discovered that incorporating silver nanoparticles resulted in a noteworthy enhancement in the luminescence intensity. They proposed that the enhancement was due to energy transfer from the nanoparticles to the REIs. For their capacity to enhance the rare earth ions' environment via the localized surface plasmon resonance (LSPR) effect, metallic nanoparticles are prioritized over the corresponding non-metallic counterpart for luminescence optimization [23]. Moreover, gold and silver are the most researched metallic nanoparticles due to their excellent absorption in the visible region. However, their cost ineffectiveness and toxicity remain key issues [24]. CuO metallic nanoparticles, on the other hand, are characterized by cheapness, small energy gap, low toxicity, and physical-thermal stability. Yet through their superior redox reaction and coloring effects, they can optimize the up-conversion nonlinear optical properties of REIs doped systems [[25], [26], [27]]. Besides its coloring effect, the accelerated nonlinear reaction of Cu^2+^ ions (ranging from 10^−6^ to 10^−12^ s) is harnessed in optical communication systems via optical fibers [28].

Considering the limited literature reports on the Dy^3+^-doped up-conversion glass system with practical and fundamental importance, a new multi-component strontium-telluroborate glass matrix with the composition 69B_2_O_3_–20SrCO_3_–7TeO_2_–3Al_2_O_3_–1MgO was developed purposely to study the up-conversion emission of Dy^3+^ single doping. Moreover, the influence of copper oxide on the up-conversion properties was explored. As far as we know, this is the first time such a study has been carried out. We reported an extension of this work in which the effect of CuONps on the luminescence traits of Dy^3+^/Sm^3+^ co-activated samples was studied [29]. Combining metallic nanoparticles like CuO and rare earth ions (REIs) such as Dy^3+^ introduces novel complexities in comprehending the properties of these materials and their potential effect on up-conversion processes. Consequently, there is an urgent need for research to elucidate the relationship between CuO nanoparticles and the up-conversion optical characteristics of Dy^3+^ mono-doped systems and to investigate their possible advantages. Telluroborate glass host was chosen due to its unique features, as revealed in the reports of [15]. The incorporation of strontium, aluminium, and magnesium is based on their ability to enhance the nonlinear optical features, compactness/chemical durability, and prevent melt crystallization of glass networks [30,31]. On the other hand, copper oxide is relatively cheap and chemically and physically stable. Its rich redox reaction and coloring effects can influence optical properties and up-conversion emission.

## 2 Materials and methods

A series of Dy^3+^ doped multi-component strontium-telluroborate glass samples with composition (69-x)B_2_O_3_–20SrCO_3_–7TeO_2_–3Al_2_O_3_–1MgO-xDy_2_O_3_ mol% (0.6 ≤ x ≤ 1.5 mol%) was synthesized via the melt-quenching method. The optimum Dy_2_O_3_ doping composition was identified and embedded with 0.2 mol% CuO nanoparticles. The powdered starting reagents were SrCO_3_ (Sigma Aldrich of 99.9% purity), B_2_O_3_ (ThermoFisher of 98% purity), TeO_2_ (Sigma Aldrich of 99.9% purity), MgO (Sigma Aldrich of 99% purity), Dy_2_O_3_ (Sigma Aldrich of ≥99.9% purity), and high purity CuO nanoparticles (Sigma Aldrich). Each sample weighing 15 g (called batch glass) was prepared in each batch by appropriately weighing the chemicals. The rigorously mixed chemicals were placed into an alumina crucible and heated in a muffle furnace at 1100 °C for 50 min. The molten chemicals were quenched on a pre-heated steel plate and then annealed at 400 °C for 3 h. Finally, the samples were allowed to cool down to room temperature by switching off the furnace. While highly transparent colorless CuO nanoparticle-free glasses were obtained, a greenish-blue transparent CuO nanoparticles-containing glass was obtained. The constitution and codification of the developed samples are presented in Table 1. XRD analysis was carried out using Rigaku X-ray Diffractometer with serial number BD68000165-01 in the angle range of 20–80 °C. HRTEM images were recorded to examine the nucleation of CuO nanoparticles in the host matrix (HITACHI HT7700 transmission electron microscope, Model 3139–03, Japan). The FTIR analysis of the samples was done using a PerkinElmer spectrometer through a wavenumber range of 650–4000 cm^−1^. EDX-mapping of the glass samples was performed using an analytical microscope with serial number JEM-ARM200F at 200 kV operating voltage. Shimadzu 3600Plus spectrometer was used for the UV–Vis-IR absorption measurement (300–1800 nm wavelength range). Photoluminescence (PL) up-conversion emission spectra in the 300–750 nm range were obtained using a Fluoromax-4C spectrofluorometer. Fluorescence decay patterns of the glasses were recorded by a Horiba PTI spectrophotometer (QuantaMaster™ 60).

**Table 1:** Glass compositions and their codes.

Composition (mol%)	Glass code
69B_2_O_3_–20SrCO_3_–7TeO_2_–3Al_2_O_3_–1MgO	STAMB0
68.4B_2_O_3_–20SrCO_3_–7TeO_2_–3Al_2_O_3_–1MgO-0.6Dy_2_O_3_	STAMB1
68.2B_2_O_3_–20SrCO_3_–7TeO_2_–3Al_2_O_3_–1MgO-0.8Dy_2_O_3_	STAMB2
68B_2_O_3_–20SrCO_3_–7TeO_2_–3Al_2_O_3_–1MgO–1Dy_2_O_3_	STAMB3
67.8B_2_O_3_–20SrCO_3_–7TeO_2_–3Al_2_O_3_–1MgO-1.2Dy_2_O_3_	STAMB4
67.5B_2_O_3_–20SrCO_3_–7TeO_2_–3Al_2_O_3_–1MgO-1.5Dy_2_O_3_	STAMB5
68.2B_2_O_3_–20SrCO_3_–7TeO_2_–3Al_2_O_3_–1MgO-0.6Dy_2_O_3_-0.2CuO	STAMB6

## 3 Results and discussion

### 3.1 Structural properties of samples

**Fig. 1:**
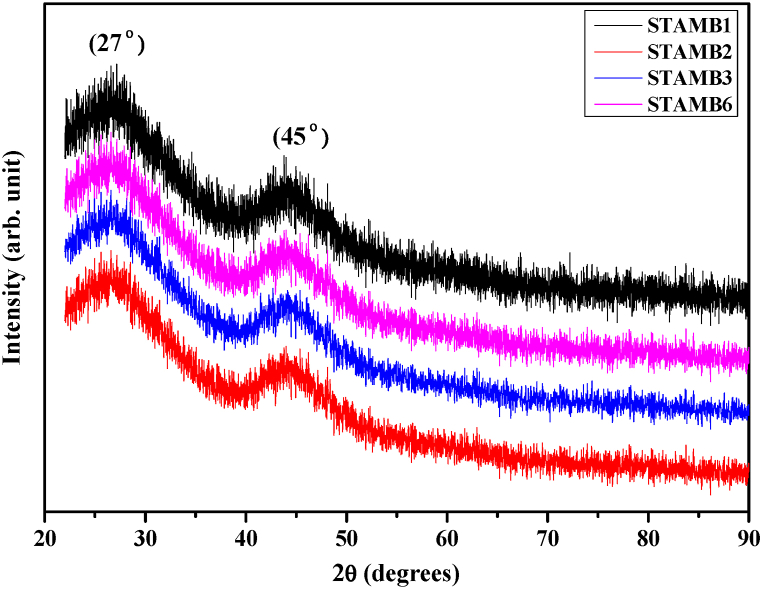
XRD pattern of the synthesized glass samples.

To assess the actual phase of the produced as-quenched systems, XRD analysis was carried out. Fig. 1 shows the XRD profile of the synthesized STAMB glass samples. The broad hunch in the pattern strongly confirmed the amorphous phase of the proposed samples.

**Fig. 2:**
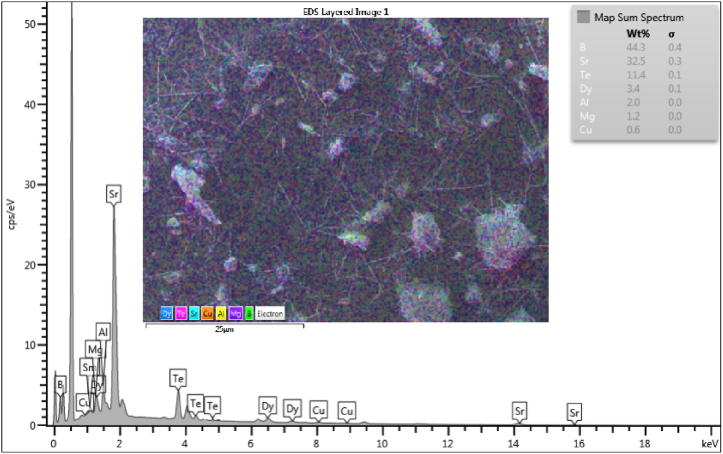
EDX spectrum of STAMB6 glass (Inset: electron layered image).

**Fig. 3:**
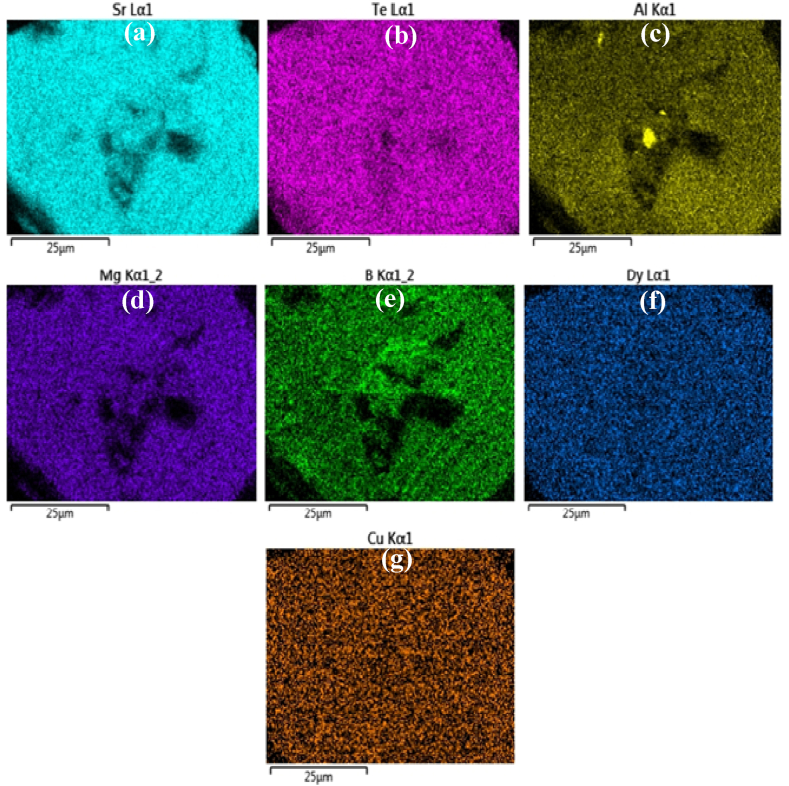
EDX maps of STAMB6 glass showing the homogeneous distribution of (a) Strontium, (b) Tellurium, (c) Aluminum, (d) Magnesium, (e) Boron, (f) Dysprosium, and (g) Copper elements, respectively.

To identify the elemental composition and homogenous distribution on elements in the synthesized glasses, EDX analysis was performed. Fig. 2, Fig. 3 depict the EDX spectrum and maps of the STAMB6 glass sample, respectively. As perceived from the micrograph, the individual starting elements were successfully incorporated. Sr, Te, and Sm were detected along *Lα* while Al, Mg, B, Dy and Cu were detected along *Kα* lines. The corresponding electron-layered image at 25 μm resolution is also shown.

**Fig. 4:**
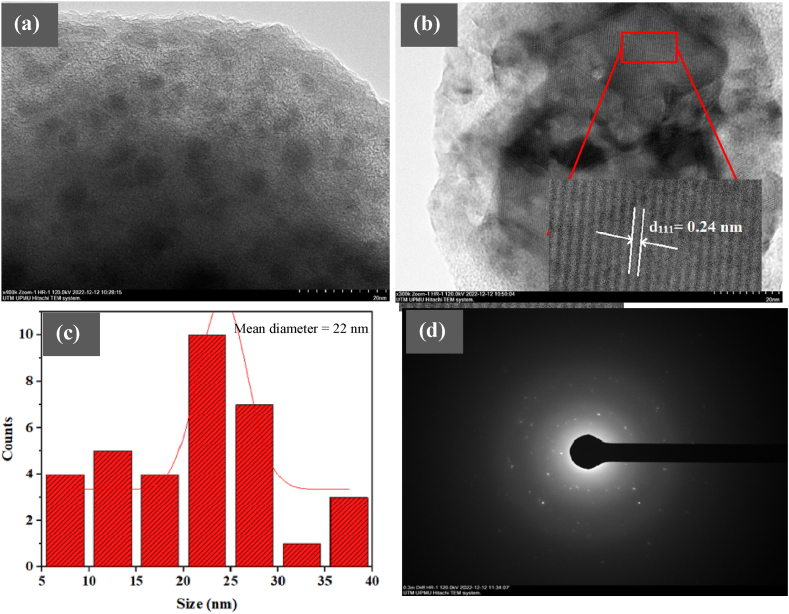
**(a)** HRTEM image of STAMB6 glass, (b) lattice fringe pattern and d-spacing of single CuO nanoparticle, (c) size distribution, and (d) diffraction ring pattern of CuO nanoparticles.

Fig. 4a shows the HRTEM image of STAMB6 glass, confirming the existence of CuO nanoparticles in the host matrix. Non-agglomerated spherical CuO nanoparticles of various sizes were dispersed in the glass matrix (black dots). Fig. 4b illustrates the lattice fringe pattern (face-centered-cubic 111 plane of Cu lattice) and corresponding d-spacing (0.24 nm) of an isolated CuO nanoparticle [32]. Fig. 4c displays the size distribution of nanoparticles with a mean diameter of 22 nm, whereas Fig. 4d represents the diffraction ring pattern of the CuO nanoparticles.

**Fig. 5:**
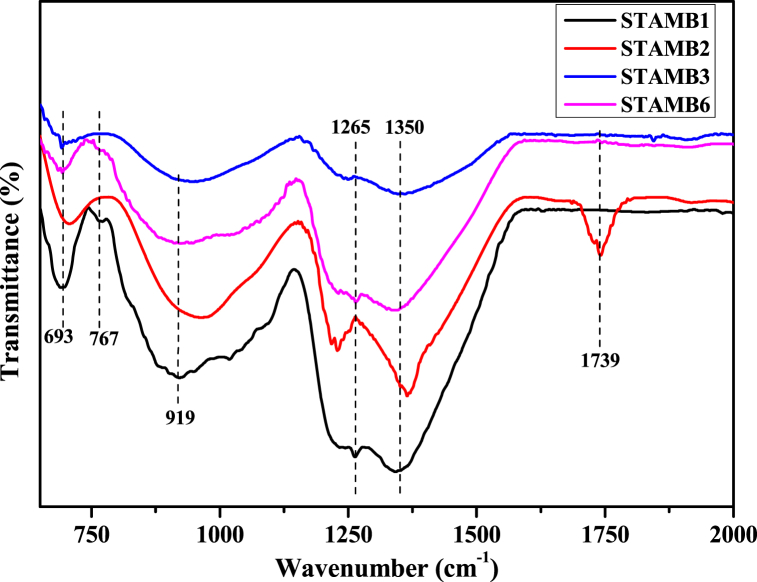
FTIR spectrum of the developed STAMB samples.

Fig. 5 displays the FTIR spectra of the prepared STAMB glasses in which different vibrational modes of the functional groups were revealed. The FTIR band traced at 693 cm^−1^ was ascribed to either the bending oscillation of the Al–O tetrahedral unit or the bending oscillation of B–*O*–B linkages, while the vibrational mode identified at 767 cm^−1^was due to the bending vibrations of BO_3_-BO_4_ units [33]. The bands at 919, 1265, and 1350 cm^−1^ are due to vibrations of different borate units in BO_3_/BO_4_ groups [33,34]. The absence of bands at around 806 cm^−1^ confirmed the absence of the boroxol ring in the glass network. The vibrational modes of the STAMB1 glass sample are more prominent than other samples. This indicated that an increase in the Dy_2_O_3_ contents could reduce the vibrations of the functional groups. Moreover, the inclusion of CuO nanoparticles into the glass sample reduced the intensity of the vibrations with the emergence of an additional peak at 1739 cm^−1^. This other peak was allotted to the vibrations of either BO_3_ or OH units or both [35]. For the CuO-free glass samples, the FTIR peak centers, configurations, and positions remained independent of the dopants concentration; a similar observation was reported by Ref. [36]. However, CuO nanoparticles caused slight shifting of the IR bands initially located at 919, 1265, and 1350 cm^−1^, respectively. The observed shift advocates the asymmetric elongation of REI-*O*-B with the conversion of BO_3_ to BO_4_ units.

### 3.2 Physical and optical properties of glasses

**Fig. 6:**
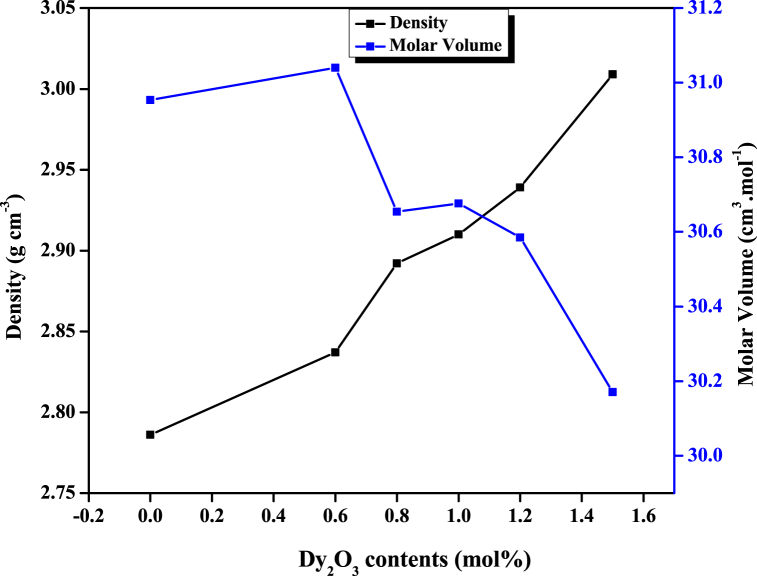
Variation of density and molar volume with Dy_2_O_3_ contents.

The physical attributes of the developed glass samples, as obtained using the formulations given in Ref. [37], were presented in Table 2. These properties are derivatives of the glass density, and their variation pattern is, thus, determined by that of the density. The density of the glasses was increased from 2.7859 to 2.837 g cm^−3^ with the increase of Dy_2_O_3_ contents. The observed increase in the density was linked to the denser nature of Dy_2_O_3_ (7.8 g cm^−3^) compared with the replaced B_2_O_3_ (2.46 g cm^−3^). In agreement with other reports [38], a similar situation was observed with the introduction of CuO nanoparticles (6.31 g cm^−3^) into the glass system, wherein the density of the STAMB1 sample increased from 2.84 to 2.93 g cm^−3^ in response. Fig. 6 shows the variation of the glass density and molar volume with Dy_2_O_3_ contents. The observed density variation of the developed glasses indicates the adjustments in the corresponding structural attributes mediated by the changes in the coordination, geometrical configuration, and interstitial holes [39]. The molar volume also increased from 30.953 cm^3^ mol^−1^ to 31.04 cm^3^ mol^−1^ in response to the initial introduction of Dy_2_O_3_ dopant and decreased continuously with increasing Dy2O3 contents reported by Ref. [40]. The molar volume enhancement was linked to the elongation of the Dy–O bond within the glass samples [37]. The witnessed density and molar volume increase suggest an enhancement in the rigidness and compactness of the glass samples [41]. While the polaron radius exhibited a reduction pattern in response to the increase in Dy_2_O_3_ content, the field strength on one side displayed a growing trend. These attest to the structural readjustments within the glass network induced by the Dy_2_O_3_ dopant [42].

**Table 2:** Physical and optical properties of the prepared glass samples.

Physical parameters	Glass samples						
	STAMB0	STAMB1	STAMB2	STAMB3	STAMB4	STAMB5	STAMB6
Density ρ, (±0.002 g cm^−3^)	2.786	2.837	2.892	2.910	2.939	3.009	2.934
Molar volume, V_m_ (±0.022 cm^3^ mol^−1^)	30.953	31.040	30.654	30.676	30.585	30.171	30.011
Ionic conc., N (±0.003 × 10^22^ ions.cm^−3^)	0.000	1.164	1.572	1.963	2.363	2.994	1.204
Polaron radius, r_p_ (±0.001 × 10^−10^ m)	–	1.778	1.609	1.494	1.404	1.298	1.758
Field strength, F (±0.004 × 10^17^ cm^−2^)	–	2.087	2.550	2.957	3.346	3.918	2.135
Inter-nuclear distance, r_i_ (±0.00 × 0^−10^ m)	–	4.412	3.992	3.707	3.485	3.220	4.363
Direct Bandgap, E_dir_ (±0.363 eV)	3.935	3.938	3.943	3.947	3.955	3.956	3.060
Indirect Bandgap, E_ind_ (±0.120 eV)	3.589	3.590	3.607	3.624	3.658	3.639	2.607
Refractive index, n (±0.001)	2.255	2.254	2.251	2.247	2.239	2.243	2.512
Molar refractivity, R_m_ (±0.05 cm^3^ mol^−1^)	17.840	17.889	17.636	17.618	17.505	17.301	19.174
Electr. polarizability,α_m_ (±0.03 cm^3^ mol)	7.079	7.099	6.999	6.991	6.947	6.865	7.609
Dielectric constant, *ε* (±0.01)	5.081	5.080	5.065	5.048	5.015	5.033	6.308

**Fig. 7:**
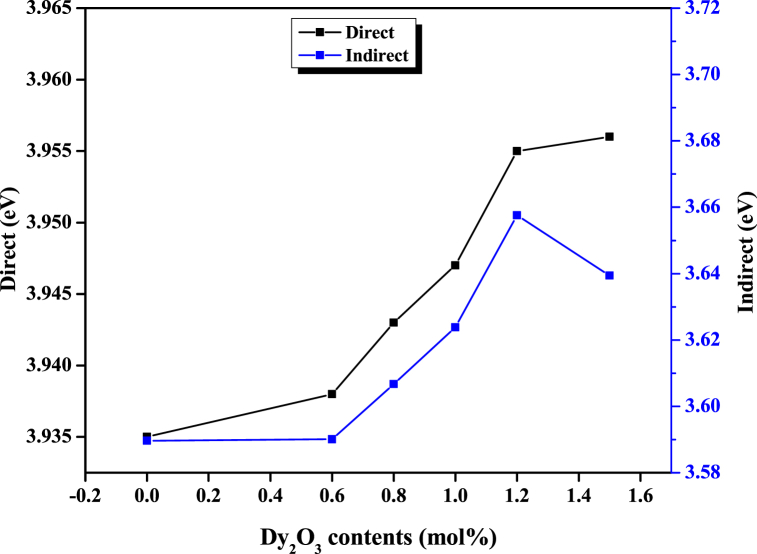
Variation of direct and indirect bandgap energies with Dy_2_O_3_ contents.

Bandgap energy (E_g_) plays a vital role in determining the optical properties of materials [43]. Thus, other optical properties of the glass samples herein were derived from their corresponding bandgap energies, and their variation is responsive to the variation of the bandgap energies. Both the direct (E_dir_) and indirect (E_ind_) bandgap energies associated with the fabricated glass samples were determined from the absorption spectra using Tauc plot [44] and are presented in Table 2. While for direct bandgap energies, the lowest level of the momentum vector in the conduction band matches the highest level in the valence band, the reverse is the case for the indirect bandgap energies. The obtained enormous bandgap energy (>3 eV for all samples) affirmed the insulating nature of the developed glasses. As elucidated in Fig. 7, the bandgap energies were found to increase slightly with the increase in Dy_2_O_3_ contents, a consequence of fundamental edge shifting due to the formation of BO_4_ from BO_3_ units prompted by the rise in the concentration of the Dy_2_O_3_ dopant in the conduction band [45]. The observed bandgap variation agrees with the reports of [26] but contrasts with the findings of [46]. The witnessed bandgap energy increase enhances the transparency (in the visible region) and insulating property of the glass systems. Incorporating CuO nanoparticles into the glass system reduced the direct and indirect bandgap energies from 3.938 to 3.59 to 3.06 and 2.607 eV, respectively. Jiménez et al. reported the same case where the bandgap energy of Erbium-doped alumino-phosphate glass reduced from 3.66 to 2.8 eV upon introducing CuO into the glass matrix [47]. The observed bandgap reduction indicates an enhancement of the up-converting ability of the glass samples. The refractive index parameter decreased slightly with increasing Dy2O3 content in response to the bandgap energy variation, as reported by Ref. [48]. As the refractive index of a glass is related to its density [49] and, therefore, its packing density, glasses with a higher refractive index tend to have a more tightly packed atomic structure, which can increase their bond strength. Thus, the decrease in the refractive index indicates a reduction in the bond strength of the Dy–O network. The correlation between the rise in band gap energy and the reduction of refractive index, induced by an increase in Dy_2_O_3_ content, may be attributed to the glasses following a standard dispersion mechanism. This phenomenon could be due to structural readjustments triggered by non-bridging oxygen, leading to variations in the dielectric constant [50,51]. However, CuO nanoparticles stimulated the refractive index increase from 2.254 to 2.512. Furthermore, the variation pattern of molar refractivity matches that of electronic polarizability.

### 3.3 Optical absorption spectra of glasses

**Fig. 8:**
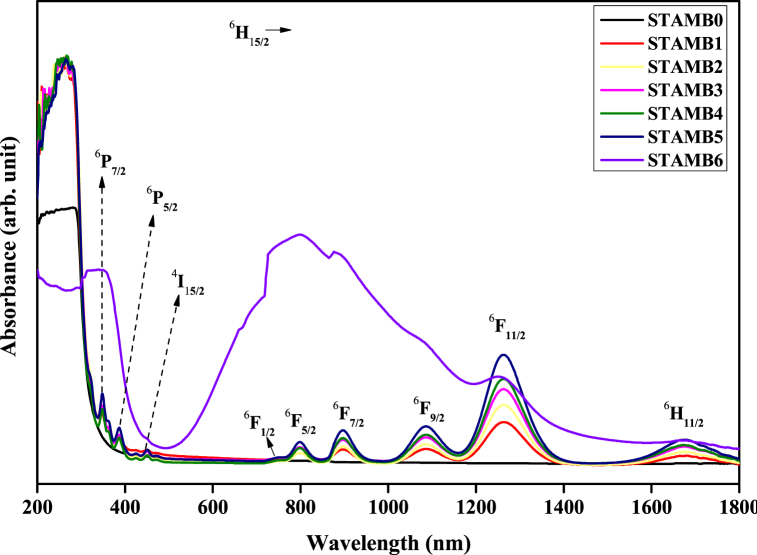
Absorption spectra of the STAMB glasses.

Fig. 8 illustrates the UV–Vis–NIR absorption spectral profiles of the synthesized STAMB glass samples. Nine characteristics of Dy^3+^ absorption transitions with peaks at 350, 386, 450, 740, 799, 897, 1084, 1261, and 1673 nm, respectively, were observed in the CuO nanoparticle-free glasses. These peaks coincided with transitions ^6^H_15/2_→: ^6^P_7/2_, ^6^P_5/2_, ^4^I_15/2_, ^6^F_1/2_, ^6^F_5/2_, ^6^F_7/2_, ^6^F_9/2_, ^6^F_11/2_, and ^6^H_11/2_, respectively [15,40]. While the peak centers and shapes remained constant irrespective of the Dy_2_O_3_ contents, the absorption intensity increases continuously with increasing Dy_2_O_3_ contents. Similar observations were reported by Abdullahi et al. [34]. Among all the observed bands, the near-infrared ^6^H_15/2_ → ^6^F_11/2_ transition band is broader and more intense than the other bands. This was linked to its hyper-sensitive nature satisfying the selection rule |ΔS| = 0, |ΔL|, |ΔJ| ≤ 2 [52]. Furthermore, no absorption transition was recorded in the visible region of 450–750 nm, linked to the impermissible spin selection rules [15]. At the higher energy absorption edge, ^6^P_7/2_ (i.e. 350 nm) transition was the strongest and thus was selected as the excitation wavelength for recording the corresponding emission spectra. Regarding the CuO nanoparticles-containing glass sample, the absorption spectrum revealed three distinct peaks matching 350 (^6^P_7/2_), 1260 (^6^F_11/2_), and 1682 (^6^H_11/2_) nm, respectively. However, additional high intense and well-broadened peak housing ^6^F_1/2_, ^6^F_5/2_, ^6^F_7/2_, and ^6^F_9/2_ transitions with center at 798 nm is visible. The absorbance of this peak is so intense that it dominated the wavelength range of 520–1400 nm (visible-near infrared region). A similar observation was reported by Refs. [53,54]. The emergence of the rich absorption band was linked to the broad absorbance of the existing Cu^2+^ ions in the glass sample, a consequence of intra-d-d transitions [47,55].

#### 3.3.1 Oscillator strengths and Judd-Ofelt (JO) analysis

The experimental (f_exp_) and theoretical (f_cal_) oscillator strengths of the Dy^3+^ absorption transitions in the produced STAMB glasses were obtained (Table 3) to examine the absorption transition probabilities. Glasses devoid of CuO nanoparticles (STAMB1 to STAMB5) showed the infrared transition (^6^H_15/2_ → ^6^F_11/2_) with the highest oscillator strength, indicating their hyper-sensitive nature. However, the oscillator strength of ^6^H_15/2_ → ^6^F_7/2_ transition for STAMB6 glass became higher (by 8-fold) than ^6^H_15/2_ → ^6^F_11/2_ with the introduction of CuO nanoparticles into the glass matrix. This indicated a change in the coordination symmetry and an increase in the covalency of the Dy^3+^ environment of the glass due to the embedment of CuO nanoparticles. Furthermore, the oscillator strengths of the glasses were found to decrease with the increase of Dy_2_O_3_ contents. STAMB1 glass showed the highest oscillator strengths among the CuO nanoparticle-free glasses. However, the oscillator strengths of the absorption transitions for STAMB6 glass were significantly higher than the STAMB1 sample, verifying the role of the CuO nanoparticles embedment in enhancing transition probabilities, thereby readjustment the symmetry of the Dy^3+^ environment. The small values of root mean square deviation $(\delta_{rms}$) verified the validity of the F_cal_ and F_exp_ fitting.

**Table 3:** Oscillator strengths of Dy^3+^ transitions in the STAMB glass samples.

Transition ^6^H_15/2_ →	Oscillator strength (× 10^−6^)											
	STAMB1		STAMB2		STAMB3		STAMB4		STAMB5		STAMB6	
	F_exp_	F_cal_	F_exp_	F_cal_	F_exp_	F_cal_	F_exp_	F_cal_	F_exp_	F_cal_	F_exp_	F_cal_
^6^P_7/2_	0.584	0.122	0.405	0.102	0.372	0.116	0.271	0.089	0.195	0.100	13.5	11.80
^6^P_5/2_	0.319	0.038	0.177	0.030	0.193	0.035	0.121	0.026	0.138	0.030		
^4^I_15/2_	0.188	0.055	0.100	0.041	0.088	0.050	0.046	0.033	0.075	0.039		
^6^F_5/2_	0.303	0.175	0.192	0.130	0.234	0.159	0.146	0.105	0.170	0.125	27.10	11.00
^6^F_7/2_	0.399	0.41	0.308	0.332	0.364	0.386	0.284	0.291	0.313	0.327	16.70	16.90
^6^F_9/2_	0.618	0.576	0.539	0.521	0.590	0.57	0.512	0.500	0.538	0.528	7.970	4.240
^6^F_11/2_	2.080	2.060	2.050	2.040	2.130	2.120	1.990	1.990	2.020	2.010	7.530	5.110
^6^H_11/2_	0.549	0.624	0.563	0.588	0.598	0.629	0.538	0.560	0.565	0.581	0.741	7.670
$\delta_{rms}$	$\pm \;0.2\times 10^{-6}$		$\pm \;0.12\times 10^{-6}$		$\pm 0.14\times 10^{-6}$		$\pm \;0.07\times 10^{-6}$		$\pm \;0.06\times 10^{-6}$		$\pm \;6.42\times 10^{-6}$	

Table 4 presents the Judd-Ofelt intensity parameters (Ω_2_, Ω_4,_ and Ω_6_) of the glass systems obtained from the experimental oscillator strengths following the Jaidass et al. [56] report. While Ω_2_ is associated with the covalency and asymmetric nature of the Dy^3+^ surroundings, Ω_4_ and Ω_6_ are identified with the stability and rigidity of the glass samples through long-range coordination. The intensity parameters for the CuO nanoparticles-free glasses followed the trend of Ω_2_˃Ω_4_˃Ω_6_ [56]. The higher value of Ω_2_ compared to the other parameters confirmed higher covalency of the Dy^3+^ environment. However, due to the activation of CuO nanoparticles in the glass host, the trend was changed to Ω_6_˃Ω_4_˃Ω_2_, wherein the value of Ω_2_ was increased from 1.29 × 10^−20^ cm^2^ to 10.8 × 10^−20^ cm^2^ (nearly 8-fold enhancement).

**Table 4:** Judd-Ofelt intensity parameters (× 10^−20^ cm^2^)of the studied glasses.

Glass Sample	Ω_2_	Ω_4_	Ω_6_	Pattern	χ=Ω4Ω6	Reference
STAMB1	1.290	0.347	0.241	Ω_2_˃Ω_4_˃Ω_6_	1.442	This work
STAMB2	1.284	0.370	0.183	Ω_2_˃Ω_4_˃Ω_6_	2.022	This work
STAMB3	1.331	0.372	0.221	Ω_2_˃Ω_4_˃Ω_6_	1.687	This work
STAMB4	1.242	0.386	0.147	Ω_2_˃Ω_4_˃Ω_6_	2.630	This work
STAMB5	1.264	0.382	0.174	Ω_2_˃Ω_4_˃Ω_6_	2.195	This work
STAMB6	10.080	12.20	12.23	Ω_6_˃Ω_4_˃Ω_2_	0.998	This work [56]
LZBSDy0.5	11.750	3.900	3.610	Ω_2_˃Ω_4_˃Ω_6_	1.080	[57]
Zinc-phosphate	2.21	0.530	0.510	Ω_2_˃Ω_4_˃Ω_6_	1.039	[58]
Dy: CNGS	4.510	2.930	0.190	Ω_2_˃Ω_4_˃Ω_6_	15.42	[59]
BBiLDy-15	0.500	0.143	0.215	Ω_2_˃Ω_6_˃Ω_4_	0.665	

Consequently, this enhancement of Ω_2_ value caused an increase in the asymmetry of the Dy^3+^ environment. A similar observation in silver nanoparticles included Eu^3^-doped sodium borate glass [60]. Essentially, the variation of Ω_4_ and Ω_6_ values improved the spectroscopic quality factor by 2.63 fold for STAMB4 glass. Nevertheless, the CuO nanoparticles inclusion was responsible for the decrease in the quality factor of STAMB1 glass from 1.442 to 0.998. It was asserted that the obtained high value of the spectroscopic quality factor is still beneficial for lasing application.

### 3.4 Up-conversion luminescence spectral analysis

**Fig. 9:**
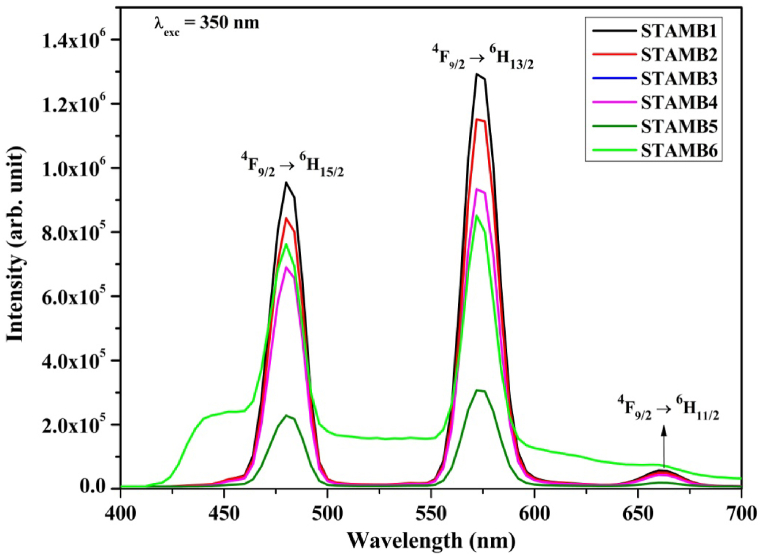
Down-conversion emission spectra of STAMB glasses.

Photoluminescence (PL) is a robust technique used to analyze the emission spectrum [61,62]. The normal down-conversion photoluminescence emission spectra of STAMB glass samples in the visible region are depicted in Fig. 9. The emission spectral acquisition was performed at 350 nm Dy^3+^ excitation wherein three distinct emission bands centered at 481,574, and 662 nm appeared. These bands matched well with transitions from ^4^F_9/2_ ground state to ^6^H_15/2_ (magnetic dipole), ^6^H_13/2_ (forced electric dipole), and ^6^H_11/2_, respectively [63]. While the magnetic dipole transition is independent of the Dy ions vicinity in the glass network, the corresponding electric dipole influences the Dy^3+^ environment. STAMB1 glass sample was found to be more intense than the other glasses; thus, it was chosen as the optimum, which agreed with the reports of [52]. The observed dousing of the emission intensity at higher Dy_2_O_3_ contents was linked to non-radiative decay arising from energy transfer through cross-relaxation processes when the Dy–Dy distances reached a critical value. It was also noted that the band centers and positions were not affected by changing the Dy_2_O_3_ contents, a similar observation reported by George et al. [64]. However, the ^6^H_13/2_ (yellow) transition was stronger than ^6^H_15/2_ (blue) due to its hypersensitive nature, and this is an indication that Dy^3+^ is located within the glass network in low symmetrical sites free of inversion centers [65]. Moreover, the influence of introducing 0.2 mol% CuO nanoparticles could be trailed by the emission spectra. While the luminescence intensity of ^6^H_15/2_ and ^6^H_13/2_ transitions decreased in response to the presence of CuO nanoparticles, that of the ^6^H_11/2_ (red) weak transition increased. However, the blue band was found to be broadened. The observed peak broadening may be ascribed to the strong absorption of the nanoparticles in the visible region as evidenced by the Uv–Vis absorption analysis cum emission of the nanoparticles at slightly different wavelengths resulting in the violet emission band at around 406 nm, a consequence of their size variations [66,67]. This observation indicates the ability of the introduced CuO to stimulate structural adjustments within the glass, thereby modifying the optical properties.

**Fig. 10:**
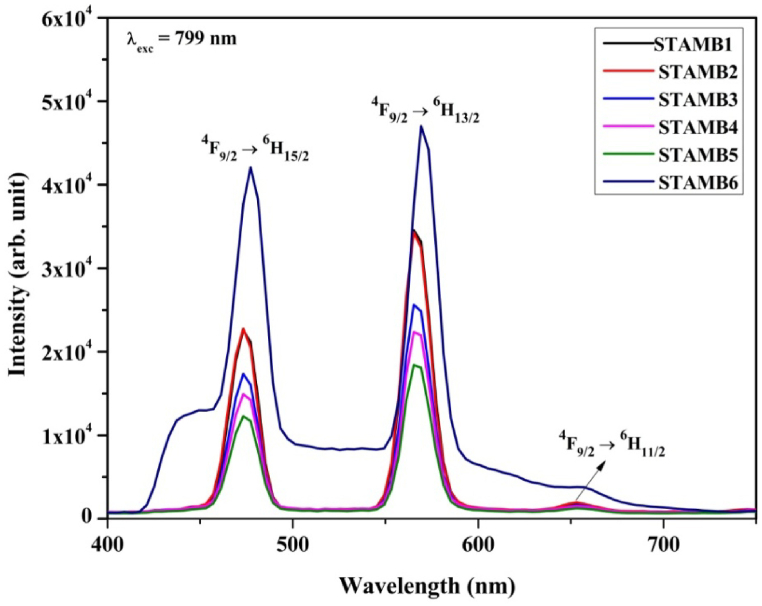
Up-conversion emission of the synthesized STAMB glasses.

Fig. 10 displays the corresponding up-conversion luminescence at 799 nm infrared excitations of Dy^3+^. A decreasing trend of the up-converted emission intensity with increased Dy^3+^ contents from 0.6 to 1.5 mol% was established. Moreover, in comparison with the down-conversion emission, the peak positions of the blue, yellow, and feeble red transitions were blue-shifted by 2, 4, and 5 nm, respectively. Although, as exhibited by the STAMB6 glass matrix, the incorporation of CuO nanoparticles into the STAMB1 glass system did not affect the peak positions, the emission intensity was increased by 1.4-fold in response. The enhancement of the up-conversion luminescence intensity may be due to symmetry distortion of the Dy^3+^ environment [68]. Thus, CuO nanoparticles as an additive in the current glass matrix enhanced the up-conversion emission and strengthened the associated nonlinear optical properties. Interestingly, the up-conversion emission intensity of the STAMB6 glass sample supersedes that of other samples, a consequence of the role played by the CuO nanoparticles. The enhancement in the emission intensity of the STAMB6 glass sample at 799 nm (up-conversion) compared with 350 nm (down-conversion) excitation agreed with the report of Elseman and Rayan [67]. Their work revealed that CuO nanoparticles experienced 10-fold luminescence enhancement when excited with 740 nm infrared energy compared with 330 nm ultraviolet energy. Moreover, the Y/B intensity ratio of the up-conversion luminescence was found to be slightly less than that of the down-conversion luminescence (1.5 against 1.4); hence the symmetry of the Dy^3+^ environment was slightly enhanced, thereby adjusting the visual luminescence color output. Consequently, the observed emission can be applied to developing a multi-colour solid-state laser. Though the intensity of the up-converted emission was found to be less than that of the corresponding down-converted emission, the up-conversion activity of Dy^3+^ doping in the developed glasses and the role of CuO nanoparticles embedment were established. As an additive in the current glass matrix, CuO enhanced the up-conversion emission and strengthened the associated nonlinear optical properties. The introduction states that up-conversion luminescence may be mediated by ESA, ETU or PA, or their combination. The mechanisms of these up-conversion mediations in the current study were proposed hereafter.

#### 3.4.1 Excited state absorption (ESA) up-conversion process

**Fig. 11:**
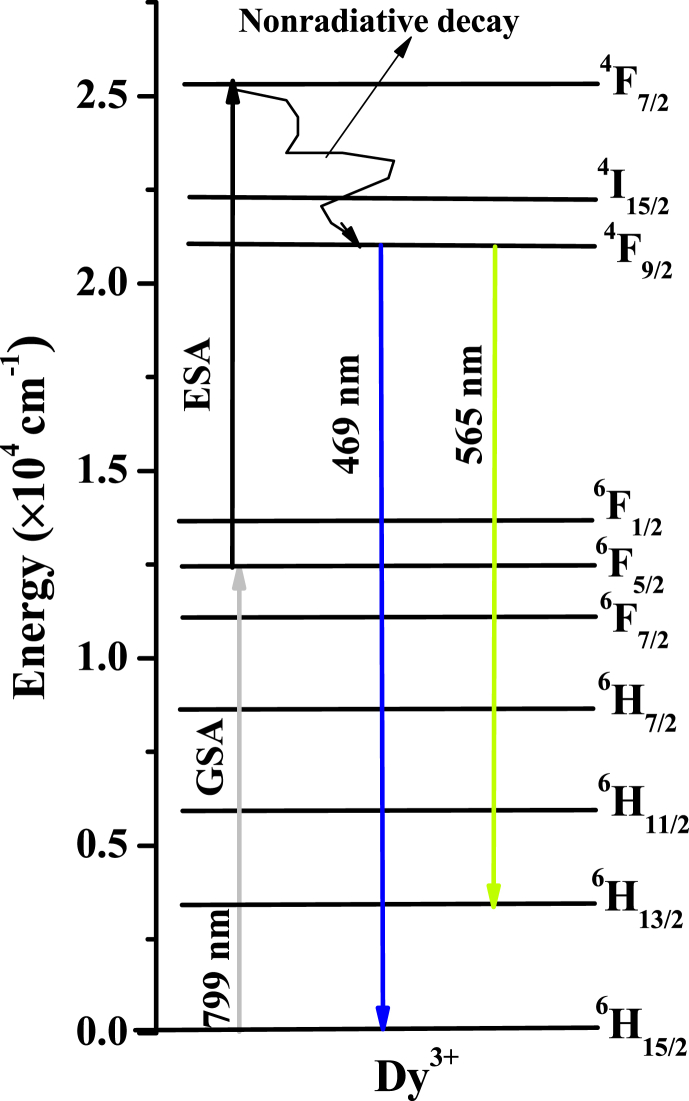
ESA-mediated up-conversion in the proposed glasses.

An energy level diagram is employed to understand the ESA up-conversion mechanism better. The ESA process involved the sequential absorption of excitation photons by a single ion from the ground state. Initially, in the ground state absorption (GSA), the excitation pumping energy 12,516 cm^−1^ (799 nm) excites Dy^3+^ ion from ^6^H_15/2_ lowest level to ^6^F_5/2_ meta-stable level. Next is the ESA step in which Dy^3+,^ already excited to ^6^F_5/2_ level, absorbs another photon from the infra-red excitation pumping energy and gets elevated to higher energy ^4^F_7/2_ excited state. However, ^4^F_7/2_ is a short-lived excited state; thus, multi-phonon interaction induces the non-radiative relaxation of the Dy^3+^ ion to the long-lived ^4^F_9/2_ meta-stable state. Moreover, owing to the significant energy difference between the populated ^4^F_9/2_ meta-stable level and the next lower-lying ^6^F_1/2_ energy level, the possibility of non-radiative multi-phonon relaxation is negligible; hence, the ion decays radiatively to the ground ^6^H_15/2_ state [69]. This results in the up-converted emission of the infrared energy input. A schematic representation of the ESA up-conversion process is shown in Fig. 11, whereas the overall process is represented as follows:

At 799 nm excitation:

1. 2^6^F_5/2_ → ^4^F_7/2_+ ^6^H_15/2_ → ^4^F_9/2_ + E_nonR_ (GSA-ESA-nonradiative decay)

2. ^4^F_9/2_ → ^6^H_15/2_ +E_R_ (yellowish + bluish emission)

#### 3.4.2 Energy transfer up-conversion (ETU) process

**Fig. 12:**
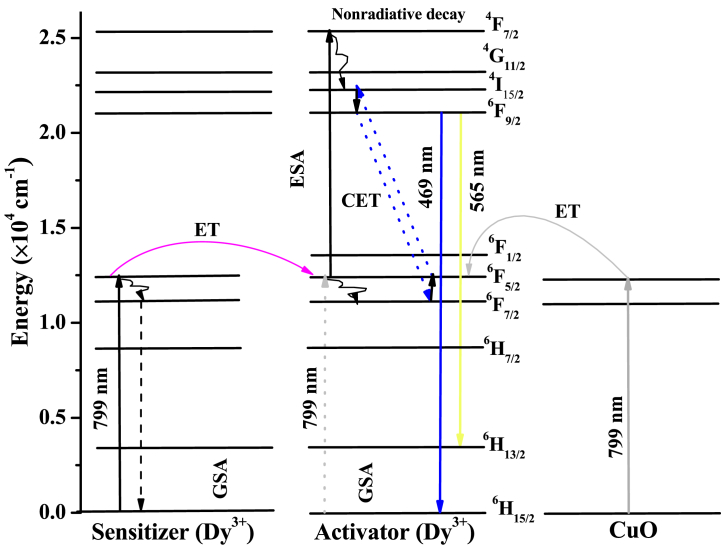
ETU-mediated luminescence in the developed STAMB glasses.

In the ETU up-conversion process, two coupled RE-ions were simultaneously excited to an intermediate excited energy level, after which one of the ions relaxed to the ground state by transferring its energy to the neighboring ion. The receiving ion is then elevated to a higher excited state, which decays radiatively by emitting up-converted luminescence. The donor ion is known as the sensitizer, whereas the receiving ion is the activator. Herein, Dy^3+^ ions at the ^6^H_15/2_ ground state are excited to the ^6^F_5/2_ state by a 799 nm excitation pump. At this level, the sensitizer ion transfers its energy to the activator ion and decays non-radiatively to the ^6^F_7/2_ intermediate level. The activator ion already at the ^6^F_5/2_ level receives the transferred energy and gets excited to the ^4^F_7/2_ varying level; however, to achieve higher stability, the ion decay non-radiatively to^4^I_15/2_ intermediate level through the ^4^G_11/2_ state. Due to instability at the ^4^I_15/2_ level coupled with energy match (≈1305 cm^−1^), the activator Dy^3+^ transfers its energy cooperatively to the Dy sensitizer ion initially relaxed to ^6^F_7/2_ state (≈1319 cm^−1^). Consequently, the sensitizer and the activator ion populate the ^4^F_S_ meta-stable level, hence intense up-conversion emission. This energy transfer process is known as cooperative energy transfer (CET) and is depicted in Fig. 12.

The intensity of ETU process-mediated luminescence indicates its efficiency and thus depends on the inter-ionic distance, a function of the Dy^3+^ concentration. It is observable from Fig. 10 that the up-conversion luminescence intensity decreases with an increase in Dy_2_O_3_ contents from 0.6 up to 1.5 mol%. The decrease in the intensity was ascribed to the quenching effect arising from cross-relaxation energy transfer [70]. Nonetheless, the up-conversion intensity increased drastically with the inclusion of CuO nanoparticles in the glass constituents. This may be attributed to either energy transfer from CuO nanoparticles to Dy^3+^, local field enhancement, or both. Although the lifetime of CuO nanoparticles (≈10^−9^ s) [71,72] is much smaller than that of Dy^3+^ (≈10^−6^ s), rendering small CuO → Dy^3+^ energy transfer efficiency, energy transfer still plays a crucial role in the observed up-conversion emission. This is due to the strong absorption of CuO nanoparticles in the visible-infrared region (as seen in Fig. 8), encompassing the 799 nm excitation energy. Again, since CuO nanoparticles absorbs and decays radiatively in the visible region by emitting light photons, herein, the emitted light photons increase the density of the available photons in the vicinity of the Dy^3+,^ and this amplified the radiative emission of the Dy^3+^ ions; consequently, the emission intensity increased drastically. Thus, it was concluded that the increased up-conversion luminescence intensity in response to the inclusion of CuO nanoparticles is due to energy transfer and local field enhancement. Fig. 12 depicts the ETU mechanism, and the probable energy transfer channels are as follows:

At 799 nm excitation:

1. ^6^F_5/2_ (Dy)+ ^6^F_5/2_ (Dy)→ 2^4^F_7/2_ (Dy) →^4^I_15/2_+ ^6^H_15/2_ → ^4^F_9/2_ + E_nonR_

2. ^4^F_9/2 →_^6^H_15/2_ +E_R_ (yellowish + bluish emission)

**Fig. 13:**
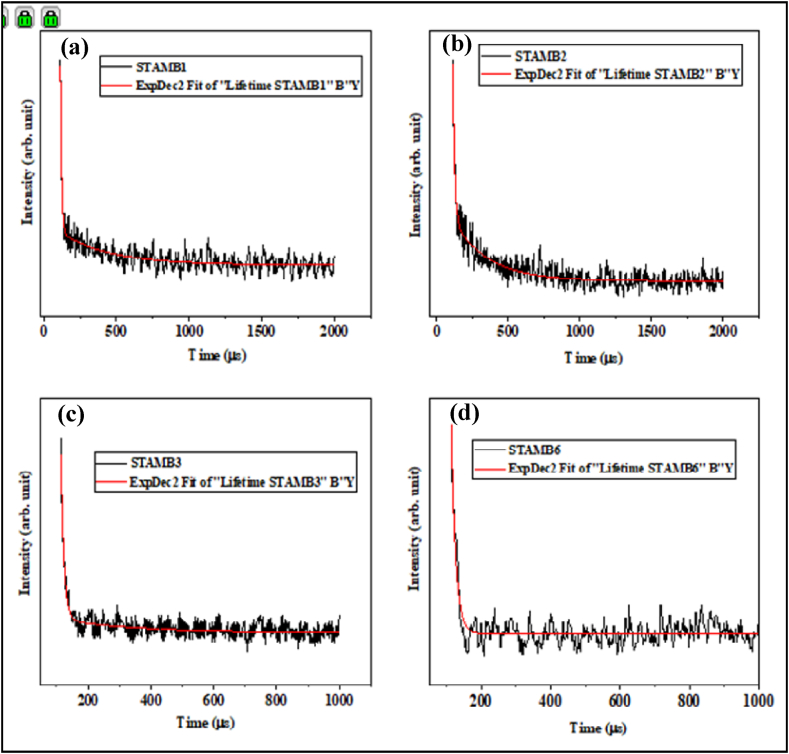
Decay profiles of (a) STAMB1, (b) STAMB2, (c) STAMB3, and (d) STAMB6 glass samples, respectively.

Radiative decay analysis was performed to explore further the ETU up-conversion mediation process in the investigated glasses. The decay patterns of the most prominent Dy^3+^ emission of the synthesized STAMB glasses are depicted in Fig. 13. All the glass samples exhibited double-exponential decay functions. Thus, the average decay lifetimes were obtained using double-exponential decay fittings. Table 5 presents the values of the obtained lifetimes from which the corresponding value of transfer efficiency (η) was found. The decay lifetime of STAMB1, STAMB2, STAMB3, and STAMB4 glass systems was found to be 9.1, 13.5, 16, and 9.7 μs, respectively. The increase in the decay lifetime of ^6^F_9/2_ level of Dy with increasing mol% contents confirmed the existence of energy transfer between Dy ions. The inclusion of CuO nanoparticles in the glass sample was found to cause an increase in the lifetime from 9.1 to 13 μs, reflecting an almost 43% increase. The perceived increase in the lifetime in response to CuO nanoparticles suggests energy transfer from CuO nanoparticles to Dy^3+^. Furthermore, the achieved energy transfer efficiency of 49% once again confirmed the mediation of energy transfer in the up-conversion luminescence revealed herein.

**Table 5:** Decay lifetimes of STAMB glass samples with the energy transfer efficiency.

Glass system	Τ (μs)	η (%)
STAMB1	9.1	20
STAMB2	13.5	17
STAMB3	16.0	21
STAMB4	9.7	16
STAMB6	13.0	49

#### 3.4.3 Photon avalanche (PA) mechanism in up-conversion luminescence

The basic requirements of PA up-conversion mediation are: substantial build-up time of the emitting energy level, the amount of participating photons should be large, a small decay lifetime, and a decreasing pattern of build-up time with the laser power [73]. As the build-up time of an activator ion is directly related to the decay lifetime of the corresponding sensitizer ion [74], the build-up time herein was found to be 0.4 ms, a value so high that the PA process is favored. Nevertheless, the number of participating photons found in this study is too low to promote the PA up-conversion process; again, the obtained decay lifetime of the Dy^3+^ activator is too large to support the PA up-conversion process. Since it is only one of the basic requirements of the PA up-conversion process is met, the possibility of PA mediation herein was ruled out. Hence, the observed up-conversion luminescence is purely mediated by ESA and ETU processes.

#### 3.4.4 Radiative properties

To unravel more, the influence of Dy_2_O_3_ contents variation cum CuO nanoparticles embedment on both the down and up-conversion luminescence of the current glass systems and radiative properties were assessed using the JO parameters and presented in Table 6. For all the developed glass samples, the radiative properties of ^4^F_9/2_ → ^6^H_13/2_ (yellow) transition dominate, and thus, it is considered for discussion. Branching ratio (β_R_), a ratio of the radiative transition probability of a particular transition to that of all transitions, is an essential property for lasing application, with ≥ 50% being the optimal value. The values obtained herein for the down-conversion emission lie in the range of 90.9%–93.1%, corresponding to STAMB1 and STAMB4 glass samples, respectively. The β_R_ is higher than that of Dy^3+^ doped lead oxy-fluoro-borate glass and Gd_3_Ga_3_Al_2_O_12_ crystal [40,75]. The higher branching ratio stressed the suitability of ^4^F_9/2_ → ^6^H_13/2_ transition for yellow lasing application. Though introducing CuO nanoparticles attenuated the β_R_ value from 90.9 to 66.9%, the value is still enough for the said application. Stimulated emission cross-section (ẟ_E_), a property that describes the probability of the existence of an excited Dy ion in a unit cross-sectional area capable of emitting a photon, is another fundamental radiative property. The ẟ_E_ value obtained for the chosen yellow transition increased from 102.4 × 10^−23^ cm^2^ to 107.2 × 10^−23^ cm^2^ with an increase in Dy_2_O_3_ content from 0.6 up to 1.0 mol% and then decreased to 100.9 × 10^−23^ cm^2^ with further Dy mol% increase. The variation in the ẟ_E_ values points to the changing probability of the existence of excited Dy^3+^ capable of emitting photons and thus varying emission intensity as confirmed from the PL spectra. The values obtained here supersede that of dysprosium ions doped bismuth borate glass [59]. Moreover, it is worth noting that the existence of CuO nanoparticles triggered the enhancement of ẟ_E_ value from 102.4 to 1301.1 × 10^−23^ cm^2^ (10-fold increase); this confirms the role of the embedded nanoparticles in strengthening the emission cross-section. While the gain bandwidth (ẟ_E_ × FWHM) was found to vary only slightly with the increase in Dy_2_O_3_ contents, the introduction of CuO nanoparticles resulted in a more than 10-fold increase in response. This once again affirmed the nanoparticles' role in improving the glass system's amplification ability. However, the fall in the magnitudes of the radiative lifetime ($\tau_{R}$) and optical gain (${ẟ}_{E}\times \tau_{R}$) confirmed the trade-off between the radiative properties in response to the CuO nanoparticles.

**Table 6:** Obtained up-conversion (upc) and down-conversion (dwc) radiative parameters.

Transition	β_R_ (%)		ẟ_E_ ( × 10^−23^ cm^2^)		ẟ_E_ × FWHM ( × 10^−28^ cm^3^)		ẟ_E_ × **τ**_R_ ( × 10^−25^ cm^2^ s^−1^)		**τ**_R_ (ms)	
	dwc	upc	dwc	upc	dwc	upc	dwc	upc	dwc	upc
STAMB1										
^4^F_9/2_ → ^6^H_15/2_	5.5	6.2	5.4	5.6	1.0	1.0	0.7	0.7	1.25	1.22
^4^F_9/2_ → ^6^H_13/2_	90.9	90.7	102.4	114.5	20.5	20.3	12.8	14.0		
^4^F_9/2_ → ^6^H_11/2_	3.6	3.1	3.3	3.1	3.4	3.8	3.1	3.0		
STAMB2										
^4^F_9/2_ → ^6^H_15/2_	4.6	4.7	4.5	4.6	0.8	0.8	0.6	0.6	1.30	1.28
^4^F_9/2_ → ^6^H_13/2_	92.2	92.2	105.9	113.5	20.0	19.8	13.8	14.5		
^4^F_9/2_ → ^6^H_11/2_	3.2	3.1	5.0	5.7	1.3	1.4	2.4	2.8		
STAMB3										
^4^F_9/2_ → ^6^H_15/2_	5.6	5.6	5.1	5.3	1.0	1.0	0.6	0.7	1.26	1.23
^4^F_9/2_ → ^6^H_13/2_	91.4	91.4	107.2	116.7	20.6	20.4	13.5	14.4		
^4^F_9/2_ → ^6^H_11/2_	3.0	3.0	5.2	5.5	1.6	2.0	1.9	2.2		
STAMB4										
^4^F_9/2_ → ^6^H_15/2_	4.1	4.1	3.8	3.8	0.7	0.7	0.5	0.5	1.38	1.32
^4^F_9/2_ → ^6^H_13/2_	93.1	93.0	100.9	106.8	19.4	19.0	13.9	14.1		
^4^F_9/2_ → ^6^H_11/2_	2.8	2.9	5.7	6.1	3.5	3.4	2.2	2.3		
STAMB5										
^4^F_9/2_ → ^6^H_15/2_	4.8	4.9	4.3	4.3	0.8	0.8	0.6	0.6	1.34	1.29
^4^F_9/2_ → ^6^H_13/2_	92.4	92.3	102.2	108.2	19.7	19.2	13.7	13.9		
^4^F_9/2_ → ^6^H_11/2_	2.8	2.8	6.7	6.2	2.1	2.2	2.9	3.0		
STAMB6										
^4^F_9/2_ → ^6^H_15/2_	25.1	26.2	274.5	275.8	60.2	59.7	1.7	1.7	0.06	0.06
^4^F_9/2_ → ^6^H_13/2_	66.9	67.0	1301.1	1309.3	247.1	244.8	8.0	7.9		
^4^F_9/2_ → ^6^H_11/2_	8.0	6.8	300.1	299.2	79.3	75.2	2.6	2.8		

For the up-conversion luminescence, the radiative properties followed the same trend as that of the down-conversion. Nevertheless, while the β_R_ and gain bandwidth values were found to be slightly less, ẟ_E_ and optical gain values were found to be somewhat higher than in the down-conversion luminescence. Similarly, the effect of CuO nanoparticles on the up-conversion emission is comparable to that in the down-conversion process.

### 3.5 CIE 1931 color chromaticity analysis

**Fig. 14:**
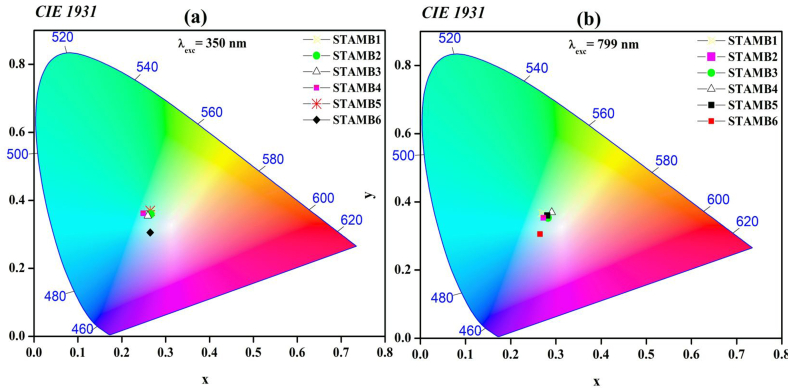
CIE 1931 chromaticity diagram of the glasses at excitation energy of (a) 350 nm and (b) 799 nm, respectively.

The accurate luminescence color display of the synthesized glasses was investigated using the 1931 CIE chromaticity guidelines [76]. The obtained CIE chromaticity diagram is shown in Fig. 14. For the normal down-conversion emission, the color coordinates of all glass samples but STAMB6 fall in the cyan zone in the cool white region; a near observation was reported by Ref. [77]. Yet, on increasing the Dy_2_O_3_ contents, the coordinates were slightly adjusted towards the pure-white region, as evidenced by the diagram. This indicates that the glass samples are suitable for white light generation. The CIE coordinates for CuO nanoparticles containing glass samples fall within the bluish-white zone with improved color purity. Hence, introducing CuO nanoparticles into the glass network can control the emission colour. The structural readjustment within the glass network is responsible for the witnessed color variability. For the up-conversion emission at 799 nm excitation wavelength, the CIE coordinates of STAMB1 to STAMB5 glasses, in accordance with the reports of [78,79], fall in the white region with high color purity (>90%). Moreover, the white color purity of STAMB6 was found to increase. Hence, at 799 nm, the glass samples are suitable as white up-conversion laser materials. The color display of the up-conversion emission was found to be a function of the excitation wavelength. The observed white color tunability in the developed glass samples was related to the Dy^3+^ local field enhancement. As the field strength is enhanced, the symmetry of the Dy^3+^ surrounding environment changes, and the covalence of the Dy–O bonds is affected. Through electron de-localization, the emission color is, thus, varied [69]. The color tuning effect of the prepared glasses revealed their suitability for application as up-conversion UV tunable laser materials.

## 4 Conclusion

Dy^3+^-activated strontium-telluro-alumino-magnesium-borate glasses were prepared and characterized to achieve an intense up-conversion photoluminescence emission. The color and intensity of the emission were found to be a function of the excitation energy. An excitation wavelength of 799 nm yielded intense up-conversion emission bands at 478 and 570 nm, respectively. The positions of the emission peaks were found to be independent of CuO nanoparticles contents, but the emission intensity was increased by 1.4-fold. In short, CuO nanoparticles embedment in the current glass matrix enhanced the up-conversion emission and strengthened the associated nonlinear optical properties. In addition, due to the influence on the evaluated JO intensity parameters, the radiative parameters of the glasses were significantly enhanced by the CuO nanoparticles. The obtained Y/B intensity ratio of the up-converted emission is more prominent than that of the down-conversion luminescence. The mediation of mechanisms like ESA and ETU in the acquired excellent luminescence characteristics of the glasses was found to be more prominent than the PA process. Thus, the mechanisms of ESA and ETU mediation processes in the observed luminescence were proposed. By this, the influence of introducing plasmonic CuO nanoparticles in the current glasses was asserted. The offered glass system may be beneficial for creating multi-color up-converted solid-state laser.

## Author contribution statement

I. Abdullahi: Conceived and designed the experiments; Performed the experiments; Analyzed and interpreted the data; Wrote the paper. S. Hashim: Contributed reagents, materials, analysis tools or data; Analyzed and interpreted the data; Wrote the paper. M.I. Sayyed: Analyzed and interpreted the data. S.K. Ghoshal: Contributed reagents, materials, analysis tools or data; Analyzed and interpreted the data; Wrote the paper.

## Data availability statement

The authors do not have permission to share data.

## Declaration of competing interest

The authors declare that they have no known competing financial interests or personal relationships that could have appeared to influence the work reported in this paper.
